# Structural and functional analysis of aquaporin-2 mutants involved in nephrogenic diabetes insipidus

**DOI:** 10.1038/s41598-023-41616-1

**Published:** 2023-09-06

**Authors:** Carl Johan Hagströmer, Jonas Hyld Steffen, Stefan Kreida, Tamim Al-Jubair, Anna Frick, Pontus Gourdon, Susanna Törnroth-Horsefield

**Affiliations:** 1 Department of Biochemistry and Structural Biology, Lund University, Lund, Sweden; 2 Department of Biomedical Sciences, University of Copenhagen, Copenhagen, Denmark; 3 Department of Chemistry and Molecular Biology, University of Gothenburg, Gothenburg, Sweden; 4 Department of Experimental Medical Science, Lund University, Lund, Sweden

**Keywords:** X-ray crystallography, Membrane proteins, Permeation and transport, Endoplasmic reticulum

## Abstract

Aquaporins are water channels found in the cell membrane, where they allow the passage of water molecules in and out of the cells. In the kidney collecting duct, arginine vasopressin-dependent trafficking of aquaporin-2 (AQP2) fine-tunes reabsorption of water from pre-urine, allowing precise regulation of the final urine volume. Point mutations in the gene for AQP2 may disturb this process and lead to nephrogenic diabetes insipidus (NDI), whereby patients void large volumes of highly hypo-osmotic urine. In recessive NDI, mutants of AQP2 are retained in the endoplasmic reticulum due to misfolding. Here we describe the structural and functional characterization of three AQP2 mutations associated with recessive NDI: T125M and T126M, situated close to a glycosylation site and A147T in the transmembrane region. Using a proteoliposome assay, we show that all three mutants permit the transport of water. The crystal structures of T125M and T126M together with biophysical characterization of all three mutants support that they retain the native structure, but that there is a significant destabilization of A147T. Our work provides unique molecular insights into the mechanisms behind recessive NDI as well as deepens our understanding of how misfolded proteins are recognized by the ER quality control system.

## Introduction

Nephrogenic Diabetes Insipidus (NDI) is a water balance disorder that is characterized by an inability to concentrate urine in response to the antidiuretic hormone arginine vasopressin (AVP). In contrast to Central Diabetes Insipidus which is caused by reduced secretion of AVP from the pituitary gland, NDI is caused by a resistance to AVP in the distal nephron. As a result, patients with NDI void large volumes of hypo-osmotic urine (typically around 12L per day) and, if urinary losses are not compensated by fluid intake, are at high risk of developing severe dehydration and hypernatremia. NDI may be acquired or congenital, with acquired NDI being more prevalent, most commonly as a result of chronic lithium treatment in bipolar disorder^1^.

**Figure 1:**
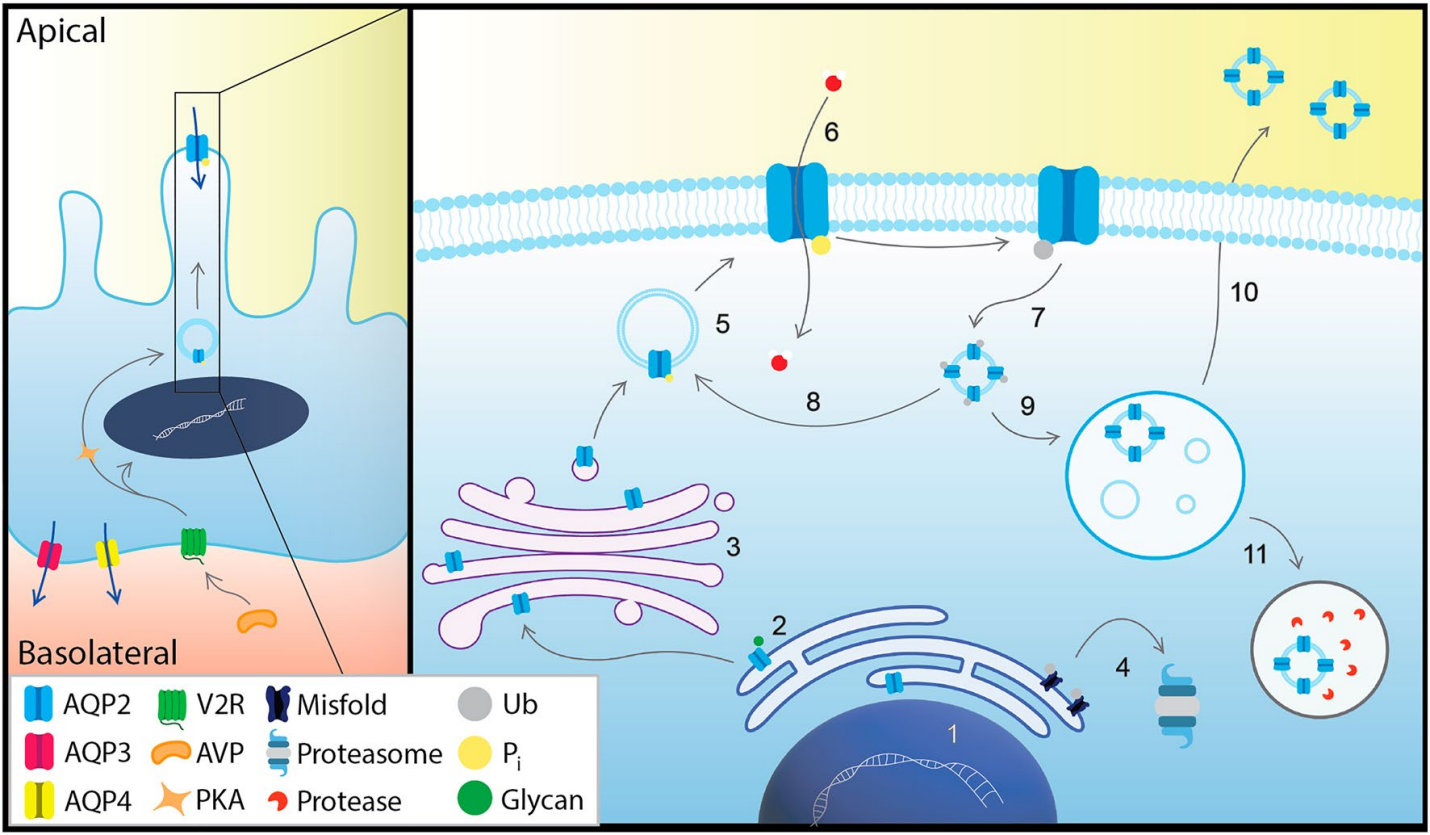
AQP2 life cycle in the collecting duct principal cells. AVP activation of V2R stimulates AQP2 expression (1), as well as PKA-dependent translocation of AQP2 stored in storage vesicles to the apical membrane. AQP2 is glycosylated co-translationally and goes through a quality control mechanism in the ER (2). Correctly folded proteins undergo further steps of glycosylation refinement in the Golgi (3), while misfolded proteins are degraded via ERAD in the proteasome (4). Once folded and glycosylated, AQP2 exits Golgi and is stored in storage vesicles where it can be phosphorylated, thereby promoting its translocation to the apical membrane (5). In the apical membrane AQP2 helps to concentrate the passing urinary filtrate by facilitating the passive transport of water molecules into the cell (6), which are eventually absorbed by the blood through transportation by AQP3 and AQP4 in the basolateral membrane. When hydration levels have been restored and the need for AQP2 is lower, changes in the AQP2 phosphorylation status and ubiquitination stimulates endocytosis (7), after which it can be either recycled (8) or relocated into MVBs (9). From there, it can be expelled via exosomes (10) or sent for lysosomal degradation (11).

The urinary concentration defect associated with NDI is a result of dysregulation of the membrane-bound water channel aquaporin 2 (AQP2) in the kidney collecting duct. Under basal conditions, AQP2 is predominantly located in subapical storage vesicles in the collecting duct principal cells^2^. Upon release from the posterior pituitary gland, AVP binds to the vasopressin V2 receptor (V2R) in the basolateral membrane, initiating a signalling cascade whereby increased cAMP-production by adenylyl cyclase stimulates Protein kinase A (PKA)-mediated phosphorylation of Ser 256 in the AQP2 C-terminus (Fig. 1). This promotes the translocation of AQP2-containing storage vesicles to the apical membrane, that, together with inhibition of endocytosis, increase AQP2 apical membrane abundance^3,4^. Phosphorylation of additional sites at Ser 264 and Thr 269 also contribute to AVP-dependent AQP2 accumulation^5–7^, as does dephosphorylation of Ser 261^8–10^. Consequently, more water is reabsorbed from the urinary filtrate and subsequently released into the hyperosmolar interstitium via AQP3 and AQP4 in the basolateral membrane^3^. Once the water balance has been restored and the AVP levels reduced, changes in the AQP2 phosphorylation status and ubiquitination of the AQP2 C-terminus stimulates its endocytosis, after which it may be stored in vesicles, targeted for degradation, or released into the urine as exosomes^11,12^. In addition, to the short-term effect on AQP2 subcellular localization, sustained increase in AVP-levels promote AQP2 gene expression via a cAMP-responsive element in the AQP2 promoter region, thereby also conferring long-term regulation of AQP2 abundance^13–17^.

**Figure 2:**
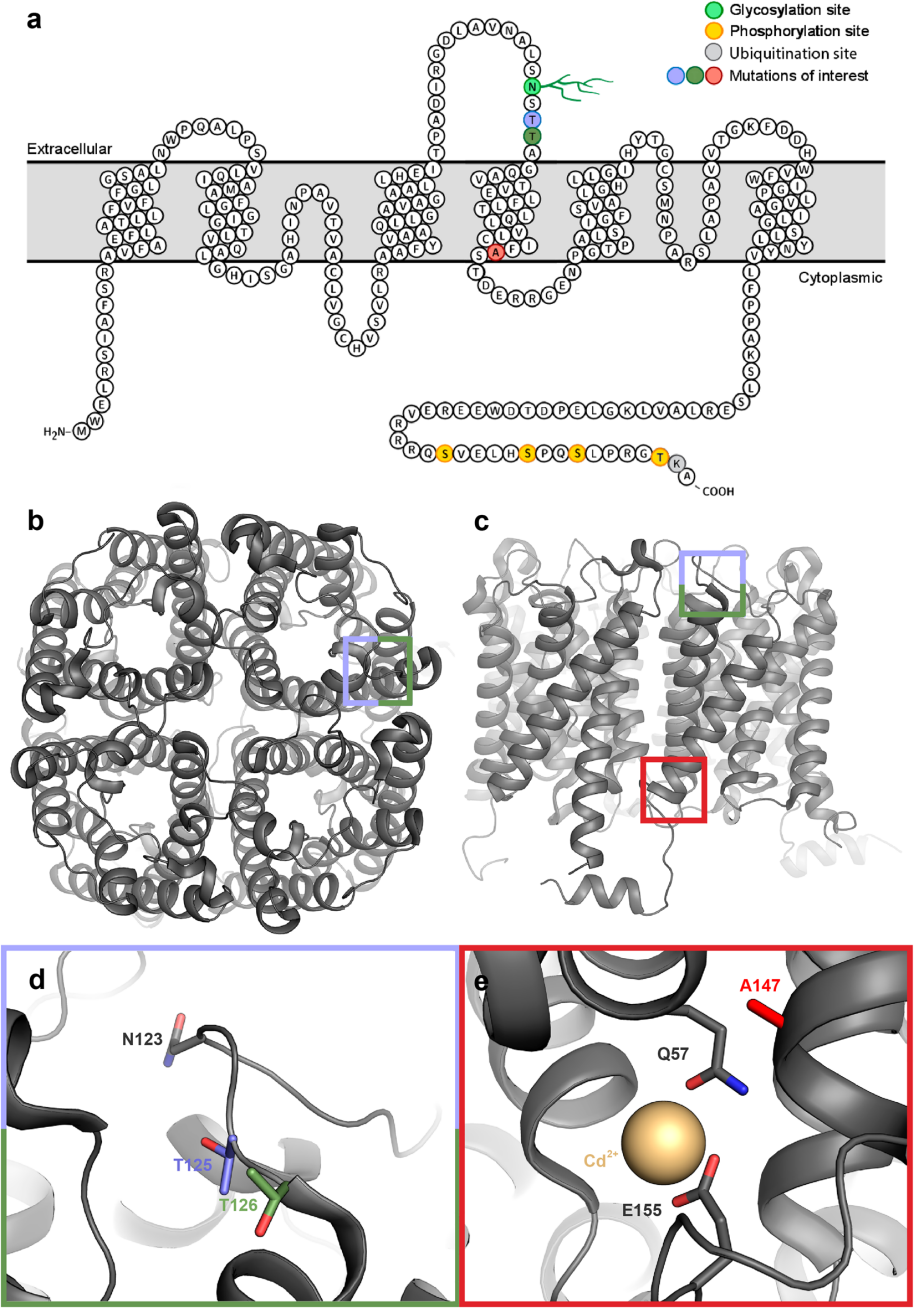
Structure of human AQP2 (**a**)Topographical representation of AQP2, with glycosylation site indicated in green, phosphorylation sites in yellow, and mutations of interest in red, blue and green. Crystal structure of human AQP2 (PDB code 4NEF) viewed from (**b**) the extracellular side and (**c**) perpendicular to the membrane. Coloured boxes indicate the location of the mutation sites T125M (blue), T126M (green) and A147T (red). (**d**) Zoom in on loop C with T125 (blue), T126 (green) and the N-glycosylation site at N123 highlighted. (**e**) Zoom in on the region around A147T mutation site with A147 highlighted in red . The Cd^2^^+^-ion present in the AQP2 crystal structure (proposed to be replaced by Ca^2^^+^ in vivo) is shown as a gold sphere and its ligands are highlighted.

Congenital NDI is typically the result of mutations in the genes for AVPR2 or AQP2. AQP2-mutations constitute approximately 10% of these and at least 70 AQP2-mutations have been identified in NDI-patients^18,19^. A majority of these mutations are inherited in an autosomal recessive fashion; however, examples of autosomal dominant inheritance also exist^20–24^. Typically, dominant mutations are found in the AQP2 C-terminus where the multiple post-translational modification sites that control AQP2 trafficking are located (Fig. 2a). Functional AQP2 assembles as a homo-tetramer (Fig. 2b and c)) and these mutants are believed to oligomerize with wild-type AQP2, forming hetero-tetramers that are disturbed in their routing to the apical membrane^21,23,24^. In contrast, recessive mutations are unable to form hetero-tetramers, most likely due to misfolding, and are instead retained in the endoplasmic reticulum (ER)^18^. These mutations can be found throughout the AQP2 six transmembrane helices and connecting loops and are proposed to affect protein folding and tetramer assembly.

To further understand the molecular mechanism behind how mutations in AQP2 cause NDI we set out to investigate the structure–function relationships of three AQP2-mutants (T125M, T126M and A147T) that have been identified in patients with autosomal recessive NDI^25,26^. T125M and T126M are located in extracellular loop C, immediately downstream to an N-glycosylation site at Asn 123 (Fig. 2d). Glycosylation at this site is believed to be important for AQP2 exit from the Golgi complex and its proper routing the plasma membrane^27^. T125M is located within the glycosylation consensus sequence (Asn-X-Ser/Thr) and this mutant can therefore not be glycosylated. Consequently, T125M is absent from the plasma membrane when expressed in oocytes^28^. In contrast, T126M has been shown to be glycosylated in oocytes^29,30^ as well as in mammalian cells^31^, but is found in a high-mannose glycosylated form that is retained in the ER where it is recognized and degraded by the ER-associated degradation pathway (ERAD). A147T is located in transmembrane helix 4 (TM4), close to a proposed Ca^2^^+^-binding site that may have implications for AQP2 trafficking^32^ (Fig. 2e). Similarly, as T126M, A147T is found in an ER-retained high-mannose glycosylated form^25,29,30^. Interestingly, the glycosylated forms of T126M and A147T were found to have significantly longer half-life than their non-glycosylated counterparts implying increased stability and/or delayed recognition by the ERAD machinery^30,31^.

Functional studies have indicated that all three mutants are able to form working water channels, suggesting that the degree of misfolding in these mutants that cause ER-retention may be minor. In oocytes the single channel water permeability (*P*_f_) for T125M, T126M and A147T was determined to be 25, 20 and 100% of the *P*_f_ for wild-type AQP2 respectively^28,29^. Moreover, stop-flow light scattering measurements on isolated ER-membrane vesicles from CHO-cells revealed that ER-retained T126M is fully functional^33^. Interestingly, chemical chaperones have been shown to promote plasma membrane targeting of both A147T and T126M, suggesting that circumvention of the ER quality control may be a treatment strategy for a subset of NDI-patients^33,34^. However, when expressed in yeast intracellular vesicles, all three mutants failed to show significant water permeability and it was argued that it is this non-functionality rather than ER-retention may be the main reason that these mutations cause NDI^35^. These conflicting results highlight the need for further studies in order to fully understand the functionality and characteristics of these mutants and their role in NDI.

In the current work, we aimed to provide the first molecular characterization of T125M, T126M and A147. For this purpose, we recombinantly expressed T125M, T126M and A147T in *Pichia pastoris* and studied their stability, water permeability and structure. Circular dichroism (CD) spectroscopy showed that all three mutants have spectra similar to wild-type AQP2, indicating that they are able to adopt the native fold. This was supported by functional studies in proteoliposomes in which all mutants permitted the transport of water. Stability measurements by CD and nanoDSF revealed that, while T125M and T126M displayed stabilities that was similar to or slightly lower than wild-type AQP2, A147T was significantly less stable. In line with this observations, all three mutants could be successfully crystallized in the same condition as wild-type AQP2, however, only T125M and T126M produced crystals of sufficient quality for structural determination. The T125M and T126M structures were solved at 3.9 Å and 3.15 Å respectively and was shown to be highly structurally similar to wild-type AQP2, indicating that the misfolding events that are recognized by the ER quality control are minor. Taken together our results provide unique molecular and structural insights into disease-causing AQP2-mutations, the implications of which for the mechanism behind their ER-retention are discussed.

## Results

### AQP2 mutants can be expressed and purified from Pichia pastoris

The T125M, T126M and A147T mutations were introduced into the plasmid used for structural determination of human AQP2^32^. This construct which contains a TEV-cleavable N-terminal His-tag and is truncated after residue 242 for crystallization purposes is referred to as wild-type AQP2 in this study. The mutants were expressed in *Pichia pastoris* and purified as previously described^32^. All mutants displayed a similar purification profile as wild-type AQP2, however the yield was lower. The final yields were ~ 5mg and ~ 2 mg pure protein per litre of fermenter culture for wild-type and T125M/T126M mutants respectively while the yield for A147T was significantly lower (< 0.5 mg).

### The A147T mutation significantly reduces protein stability

**Figure 3:**
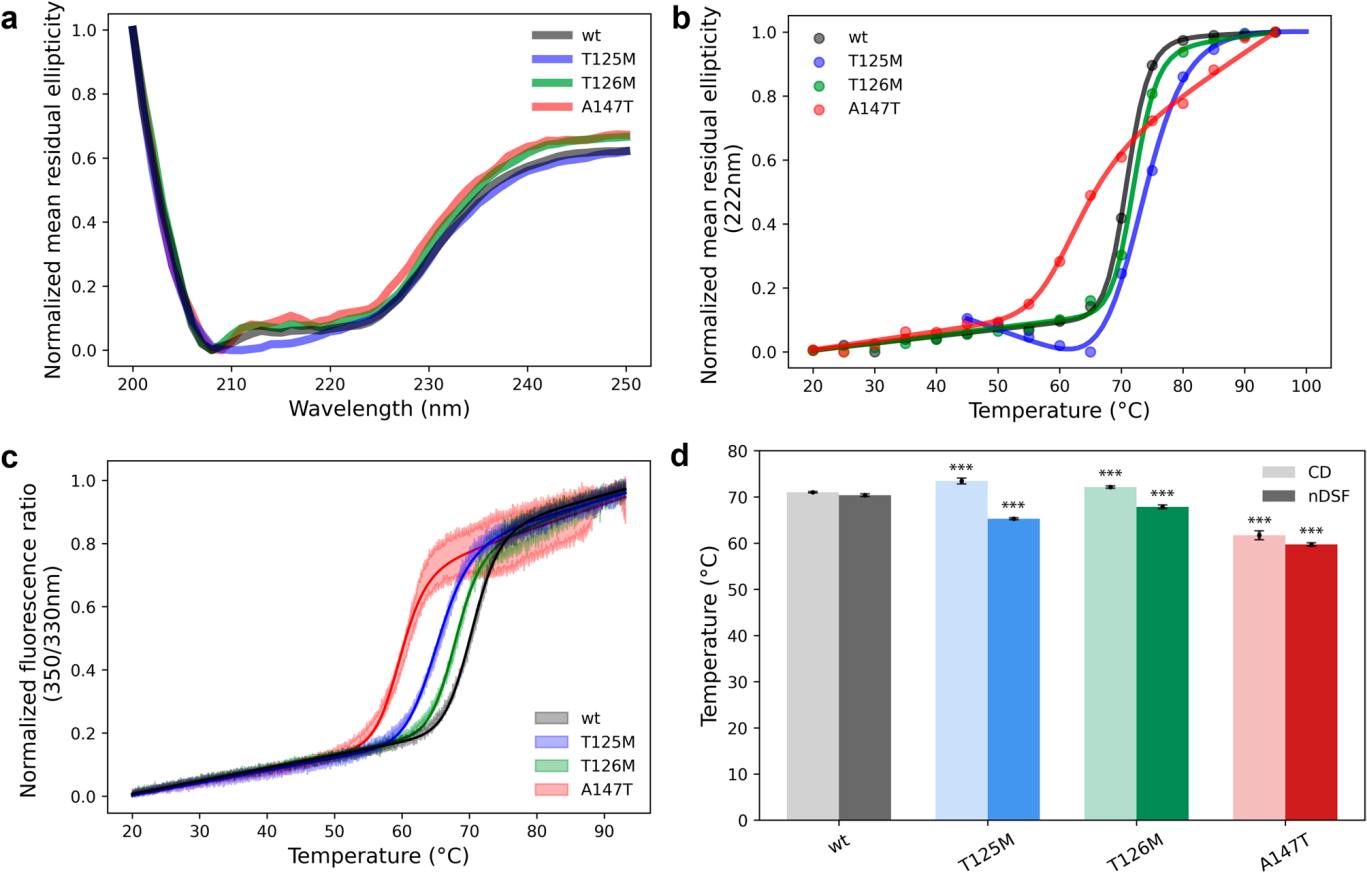
Thermal stability of wild-type AQP2 and mutants. (**a**) CD spectra showing normalized mean residual ellipticty for each constructs at 20 °C. All constructs showed the characteristic minima for alpha helical proteins at 208 nm and 222 nm. (**b**) Normalized mean residual ellipticity for each construct plotted against increasing temperature. The melting temperature (T_m_) can be estimated from the inflection point of the resulting curves. T125M, T126M and wild-type AQP2 display similar behaviour and T_m_, whereas A147T has a lower T_m_ and a more gradual transition between folded and unfolded states. (**c**) Melting curves from nDSF where the ratio between the normalized fluorescence at 350 and 330 nm is plotted against increasing temperature. T_m_ and T_onset_ can be estimated from the inflection point and the deviation from baseline respectively. The shaded area for each curve represents the standard deviation between triplicate runs. (**d**) Comparison of T_m_-values determined by the two methods. T125M and T126M displayed small variations in T_m_ compared to wild-type AQP whereas for A147T, a more pronounced reduction of T_m_ compared to wild-type AQP2 was observed using both methods. Error bars correspond to standard deviation between triplicate measurements and the statistical significance in relation to wild-type AQP2 is indicated by *** (*p* < 0.001), with exact p-values given in the main text and in Table S1.

The fold and stability of the three mutants as well as wild-type AQP2 were evaluated using CD spectroscopy. All constructs were measured in triplicate at 0.2 mg/ml, and the far-UV spectra (200–250 nm) were recorded, from 25 to 95 °C, with 5 °C increments. At 20 °C, wild-type AQP2 and T126M showed very similar spectra with the clear dips at 222 and 208 nm that are typically associated with alpha-helices (Fig. 3a)^36^. A similar shape of the spectra was also seen for T125M and A147T however the signal was much weaker, even after adjusting for concentration (Supplementary Fig. S1), which could indicate a higher degree of random coil in these two mutant samples. In order to investigate the thermal stability of the proteins, the normalized mean residual ellipticity (MRE) at 222 nm was plotted against the temperature and the melting temperature (T_m_) was obtained by fitting the resulting curve to a Boltzmann sigmoidal equation (Fig. 3b and Supplementary Table S1). Both T125M and T126M displayed similar melting curves as wild-type AQP2 and had slightly higher melting temperatures (*p* = 0.00015 and 0.00092); T_m_ was 71.03 ± 0.16, 73.45 ± 0.65, and 72.14 ± 0.25 °C for wild-type, T125M and T126M respectively. In contrast, the shape of melting curve for A147T was different with a gentler slope, indicating a less sharp transition between folded and unfolded states. Moreover, T_m_ was determined to be 61.71 ± 0.96 °C, markedly lower than for any of the other proteins (*p* < 0.00001).

The thermal stability was further studied using nanoDSF which measures the change in intrinsic fluorescence from tryptophan residues as they become exposed during protein unfolding. Each construct was measured in triplicate and melting curves were obtained by plotting the ratio between the normalized fluorescence at 330 and 350 nm against the temperature. T_m_ was obtained by fitting the curve as described above and the data was further analyzed with the MoltenProt software^37^ in order to extract the temperature for denaturation onset (T_onset_) (Fig. 3c, d and Supplementary Table S1). All melting curves displayed similar shapes with a single transition, suggesting the absence of long-lived folding intermediates. However T_m_ and T_onset_ varied between the constructs. Wild-type AQP2 had the highest stability (T_m_ = 70.39 ± 0.27, T_onset_ = 61.60 ±  0.41 °C), followed by T126M (T_m_ = 67.87 ± 0.39 °C, *p* < 0.00001; T_onset_ = 59.56 ± 0.35 °C, *p* < 0.00077) and T125M (T_m_ = 65.27 ± 0.21 °C, *p* < 0.00001; T_onset_ = 54.70 ± 0.21 °C, *p* < 0.00001). Similarly, as for the CD-measurements, A147T had the lowest thermal stability with a T_m_ and T_onset_ of 59.71 ± 0.34 and 51.22 ± 0.64 °C respectively (*p* < 0.00001).

### T125M, T126M and A147T are functional water channels.

**Figure 4:**
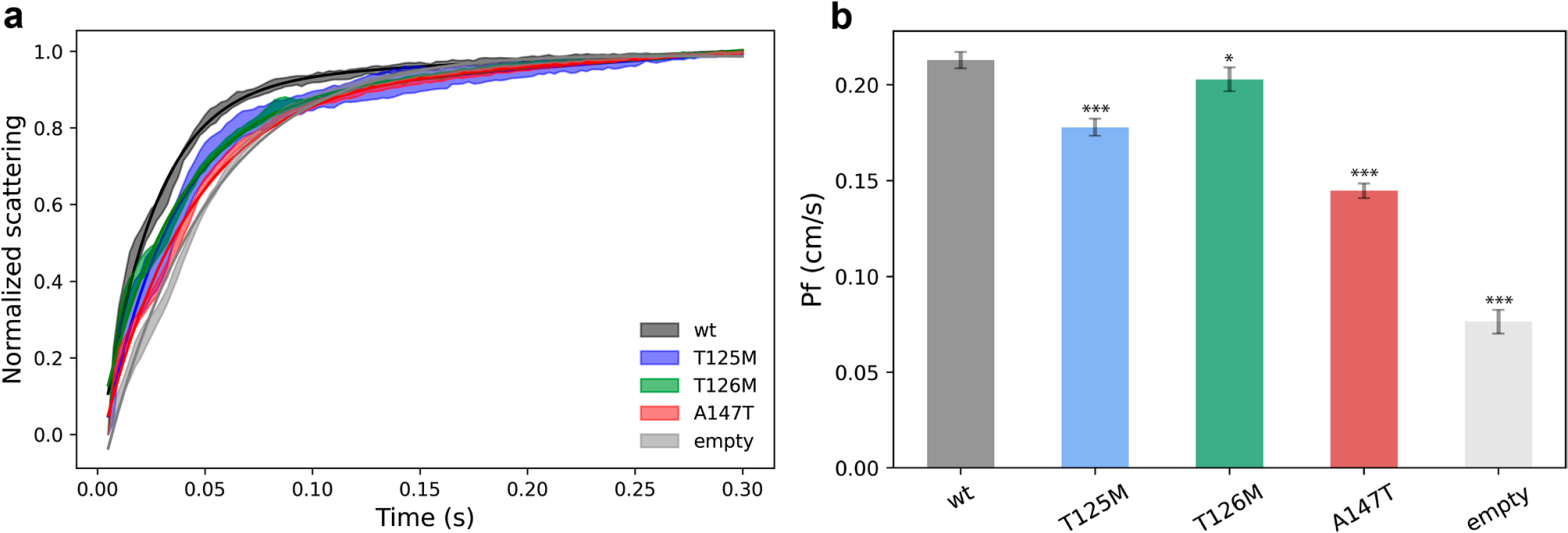
Stopped-flow measurements of water transport through AQP2 mutants (**a**) Typical normalized stopped flow scattering curves. Each curve is an average of 10 measurements. The fitted curve is showed as solid coloured lines and the standard deviation for each sample is displayed as a shaded area of the same colour. (**b**) Pf values for each construct, adjusted in relation to the reconstitution levels. Each value was calculated from three individual liposome preparations, with the exception of A147T which only could be successfully reconstituted once. All proteoliposomes showed an increase in Pf compared to empty liposomes. T126M had a slightly lower water permeability compared to wild-type AQP2 (92.6%) while the water permeability of T125M and A147T was further reduced (74.3% and 49.9% of wild-type AQP2 respectively). Error bars correspond to standard deviation from 10 measurements each from three individual proteoliposome preparations, with the exception of A147T for which only one proteoliposome preparation was used. The statistical significance is indicated by *** (*p* < 0.001) and * (*p* < 0.1) with exact *p*-values given in the main text.

In order to determine the functionality of the three mutants, T125M, T126M and A147T as well as wild-type AQP2 were reconstituted into liposomes and the water permeability was investigated using stop-flow spectroscopy (Fig. 4). Proteoliposomes and empty control liposomes carrying a fluorophore on the inside were mixed with a hyperosmotic solution, resulting in liposome shrinking that could be quantified as an increase in fluorescence. The rate constants were determined from fitting a two-exponential function to an average of 10 measurements (see Material and Methods) and used to calculate the osmotic water permeability, *Pf*. This was repeated for three independent preparations of proteoliposomes and control liposomes. The *Pf*-values were adjusted for differences in protein reconstitution levels by quantifying the amount of protein in the liposomes using a Western blot (Supplementary Fig. S2). The amount of protein in the liposomes varied significantly between the constructs and in some cases also between proteoliposome preparations of the same construct. For A147T in particular, incorporation was very low, and we could in fact only detect protein in one out of three independent liposome preparations, wherefore only this preparation was used in the analysis. This may be due to differences in tetramer stability, as observed in the size-exclusion chromatography experiment that was performed immediately prior to reconstitution in order to ensure optimal protein quality (Supplementary Fig. S3). In this experiment, wild-type AQP2 and T125M eluted mainly as tetramers with only minor contributions from other oligomeric states. In contrast, T126M and A147T both showed significant amounts (30% and 50% respectively) of a population that most likely corresponds to the monomeric states, as well as the presence of higher oligomers and aggregates. It therefore seems likely that tetramer disassembly lies behind the lower reconstitution capacity of these two mutants.

For all three mutants, as well as wild-type AQP2, the proteoliposomes displayed an increase in water permeability compared to control liposomes, showing that they are all functional water channels (Fig. 4a). The adjusted *Pf*-values (cm/s) were 0.18 ± 0.0044 for T125M, 0.20 ± 0.0062 for T126M and 0.14 ± 0.0038 for A147T, significantly higher than that obtained for control liposomes (0.076 ± 0.0062, *p* < 0.00001) (Fig. 4b). For T125M, T126M and A147T, this corresponds to 74.3% (*p* < 0.00001), 92.6% (*p* = 0.091) and 49.9% (*p* < 0.00001) water permeability of wild-type AQP2 (*Pf* = 0.2129 ± 0.0043 cm/s). These results are in agreement with studies in oocytes^28,29,38^, albeit with differences in relative *Pf* compared to wild-type AQP2, and is the first verification of direct water transport through these mutants.

### AQP2 mutant crystal quality correlates with protein stability

Hanging drop crystallization trials were set up based on the conditions for wild-type AQP2^32^ at either 4 °C or room temperature. All three mutants crystallized in these conditions, with crystals beginning to form within 15 min upon setting up the drops at the highest PEG-concentrations. The crystals were fished, and flash frozen within two to three days. If the crystals were fished after more than 4 days, they displayed a decline in diffraction quality, which would further progress into crystals giving no diffraction after 7 days.

Crystal screening at synchrotrons revealed that, despite being grown in the same conditions and having similar size and morphology, there were significant differences in diffraction quality. Overall, T126M gave crystals which diffracted to highest resolution, (~3–3.3 Å), followed by T125M (~3.7–4 Å). A147T crystals were of the lowest quality, diffracting X-rays to around 5-7 Å. This is in good agreement with the thermal stability of the three protein constructs, as determined by CD spectroscopy and nanoDSF, supporting the previously proposed correlation between protein thermal stability and ability to crystallize^39,40^.

### T125 and T126M crystal structures are highly similar to wild-type AQP2

Complete data to 3.9 Å and 3.15Å were collected for T125M and T126M respectively. The crystals belonged to the same space group as wild-type AQP2 with very similar cell dimensions (Table 1). The structures were solved by molecular replacement using the wild-type AQP2 structure as the model (PDB code 4NEF)^32^. Data was also collected to 5.0Å for A147T, however the cell parameters could not be unambiguously determined and molecular replacement was unsuccessful, most likely due to the limited resolution. Iterative model building and refinement resulted in final models with an R-factor/free R-factor of 0.295/0.312 for T125M and 0.256/0.301 for T126M. Typical electron density maps for the two structures are shown in Supplementary Figure S4.

**Table 1:** Data collection and refinement statistics.

	T125M	T126M
PDB accession code	8GHJ	8OEE
***Data collection***		
Beamline	MAXIV BioMAX	DESY P13
Wavelength	0.97658	1.033210
Space group	P4_2_	P4_2_
Cell dimensions	a = b = 118.31, c = 90.40	a = b = 118.94, c = 89.96
	*α* = *β* = *γ* = 90	*α* = *β* = *γ* = 90
Resolution*	50.0–3.90 (4.00–3.90)	89.96–3.15 Å (3.36–3.15 Å)
R_merge_*	0.198 (4.45)	0.054 (3.49)
I/*σ*I*	9.84 (0.43)	13.1 (1.1)
CC_(1/2)_*	0.999 (0.287)	0.998 (0.208)
Completeness*	99.9 (99.9)	99.80 (100.00)
Multiplicity*	21.3 (20.1)	11.3 (13.4)
***Refinement***		
No of reflections*	11,538 (1145)	42,680 (2694)
Resolution*	49.5–3.90 (3.99–3.90)	71.75–3.15 (3.22–3.15)
R_work_/R_free_*	0.296/0.312 (0.396/0.411)	0.255/0.286 (0.387/0.443)
***No of atoms***		
Protein	6842	6928
Cd	2	2
Water		15
Average B (Å^2^)		
Protein	213.43	124.06
Cd	197.27	91.84
Water		96.50
***Rms deviations***		
Bonds (Å)	0.003	0.003
Angles (°)	0.87	0.64
Ramachandran (%)		
Favoured	86.2	87.3
Allowed	13.4	12.7
Outliers	0.4	0

*Values for the highest resolution shell given in parentheses. R-free is calculated from 10 and 5% of the reflections being omitted during refinement for T125M and T126M respectively.

**Figure 5:**
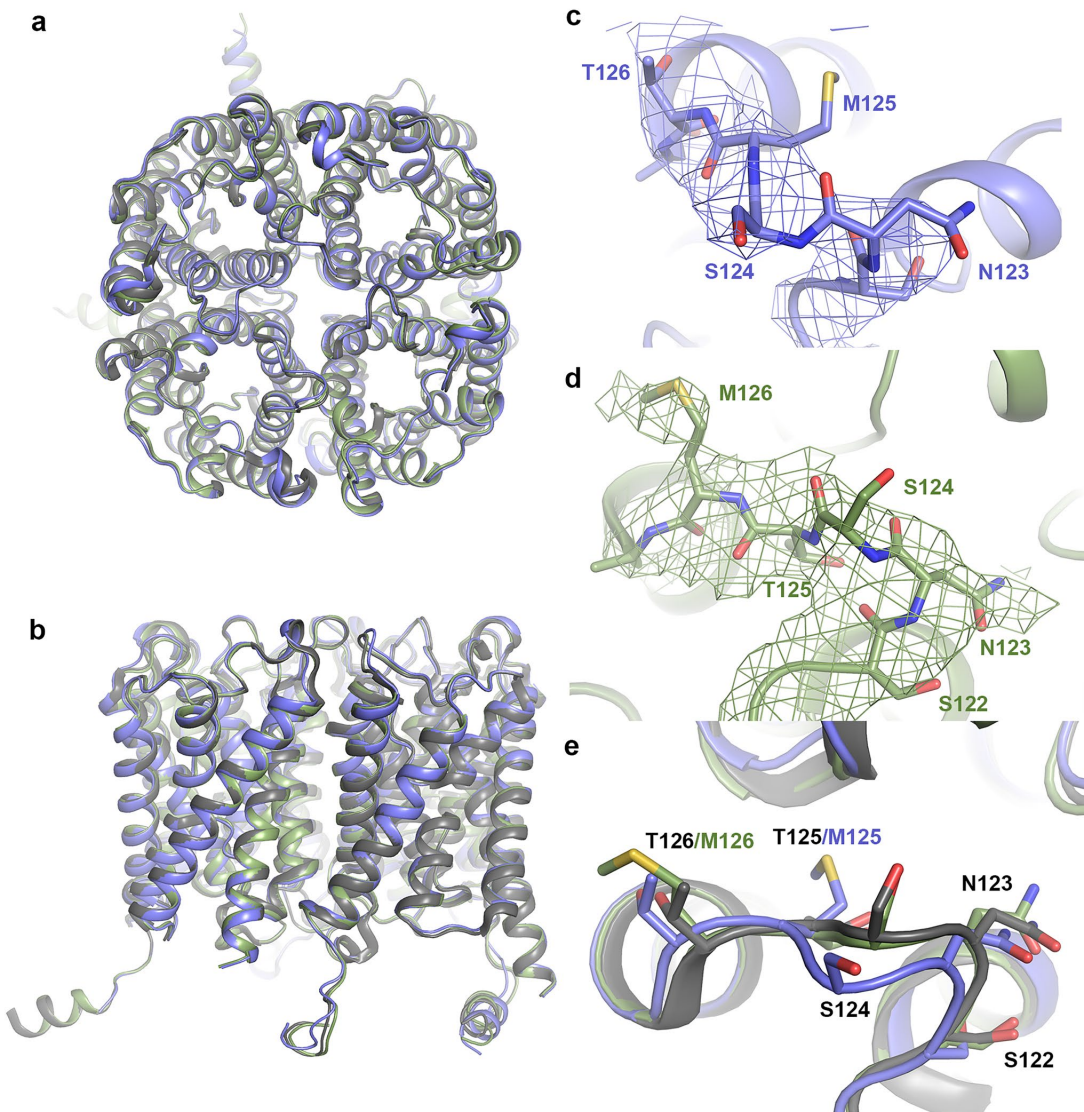
Crystal structures of T125M and T126M mutants. Overlay of wild-type AQP2 (PDB:4NEF, grey), T125M (blue) and T126M (green) viewed (**a**) from the extracellular side and. (**b**) perpendicular to the membrane illustrating the high overall structural similarity between the three structures. Zoom-in on loop C for T125M (c) and T126M (**d**). The 2F_o _− F_c_ electron density map contoured at 1.0 σ for each mutant is displayed as coloured mesh. (**e**) Overlay of loop C from wild-type AQP2 (grey), T125M (blue) and T126M (green) showing that, except for a minor displacement of the T125M main-chain around N123 and S124, the structures are very similar.

As seen in Fig. 5, the T125M and T126M-structures are highly similar to wild-type AQP2, with root-mean-square deviations (RMSD) of 0.942 Å (929 Cα-atoms) for T125M and 0.322 (940 Cα-atoms) for T126M. For both mutants, loop C which harbours the mutation sites could be modelled. Density for the methionine side chain could be observed in all monomers for T126M however this was not the case for T125M due to the lower resolution (Fig. 5c,d). Surprisingly, neither of the mutations caused any major structural perturbation of loop C or neighbouring loops. In the T125M structure, a minor displacement of the main chain could be observed around Asn 123 and Ser 124 (1–2 Å), most likely due to the larger methionine side chain. It thus seems that AQP2 can accommodate the replacement of a small polar residue with a larger hydrophobic one without any significant structural rearrangements. It should be mentioned that, in particular for T125M, the resolution is not high enough to reliably obtain information about interactions between residues and that there may be minor structural alterations regarding side-chain conformations and hydrogen bonds that we are not able to ascertain. Nevertheless, the structures clearly show that the classification of these two mutants as “misfolded” by the ER quality control must involve highly local structural changes and/or exposure of small hydrophobic patches, perhaps even single residues.

## Discussion

**Figure 6:**
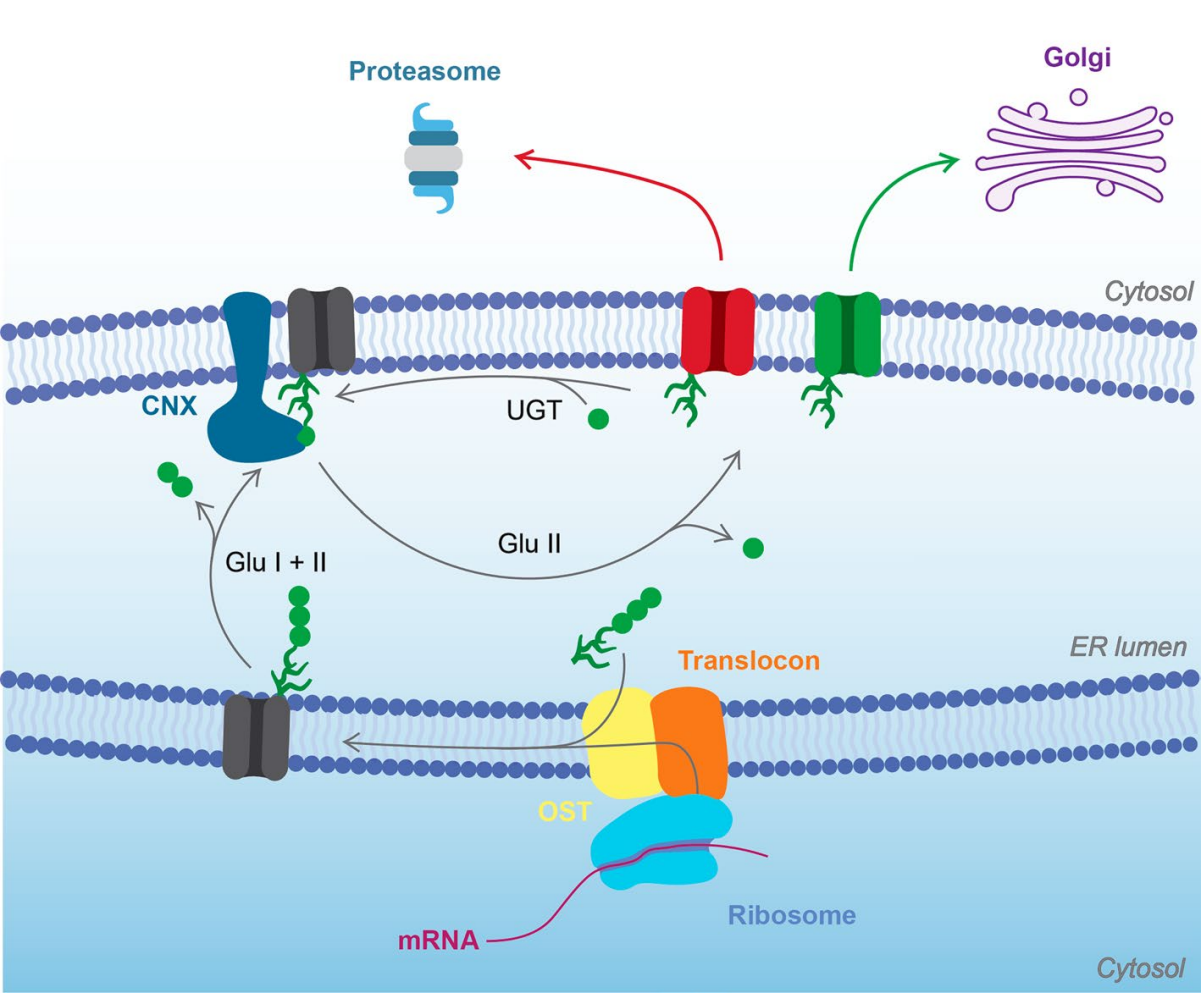
N-glycolysation-dependent quality control of membrane proteins in the ER. Membrane proteins in the secretory pathway are co-translationally N-glycosylated by oligosaccharyltransferase (OST). The attached glycan is further processed by glucosidase I and II (Glu I and Glu II) which removes to glucose units and makes the protein a substrate for the chaperone calnexin (CNX), thereby promoting its proper folding. Cleavage of the last glucose residue by Glu II releases the protein from CNX after which correctly folded proteins (green) are released to the Golgi apparatus for further glycan processing. For incorrectly folded proteins (red), the presence of misfolded domains close to the glycosylation site makes protein a substrate for UDP-glucose glycoprotein glucosyltransferase (UGT) which re-attaches a glucose residue, whereby the protein undergoes another cycle of CNX-assisted folding. This causes ER-retention and eventually, due to the action of mannosidases, the protein becomes a target for ER-associated degradation (ERAD) by the proteasome.

The ability to recognize and get rid of misfolded proteins is a fundamental cellular property that is critical for human health^41^. For newly synthesized eukaryotic proteins that are targeted to the ER for trafficking to their destination via the secretory pathway, this is achieved by a robust quality control system that allows only correctly folded proteins to exit from the ER lumen. Misfolded proteins are subjected to degradation via ERAD, whereby they are retro-translocated to the cytoplasm and degraded by the proteasome^42^. This involves a glycosylation-dependent pathway which starts with oligosaccharyltransferase (OST) co-translationally attaching a core glycan (GlcNac_2_–Man_9_–Glc_3_) to an asparagine residue^43^ (Fig. 6). Cleavage of two terminal glucose residues are by glucosidase I and II generates high affinity ligands for the lectin-based chaperones calnexin (CNX) and calreticulin (CRT) which promotes proper folding and prevents aggregation^44–46^. Finally, cleavage of the last glucose residue releases the protein from CNX/CRT after which it, if correctly folded, is transferred to Golgi where the glycan is further trimmed by mannosidases.

Proteins with non-native conformations may be reglucosylated by UDP-glucose glycoprotein glucosyltransferase (UGT), which selectively adds glucose to glycans in misfolded domains and returns them to CNX/CRT^47,48^. De-/reglucosylation are in kinetic competition with ER-mannosidases that catalyze the slow removal of mannose, decreasing the affinity towards UGT and targeting the proteins for degradation^49^. Hence, the overall system acts a folding timer, giving proteins multiple chances to fold during a limited amount of time before entering the degradative pathway^42^.

Failure to reach the plasma membrane due to being caught in the ER quality control system is believed to be the main reason why mutations in AQP2 lead to recessive NDI. This is supported by a number of studies which show that several recessive NDI-mutations, including T125M, T126M and A147T studied here, are indeed retained in the ER^25,28–31,34,50,51^. However, there is very little information about the effect of these mutations at the molecular level, wherefore the underlying reasons for ER-retention has remained elusive. Moreover, studies in oocytes and yeast vesicles have generated conflicting results regarding whether these mutants permit water transport or not^25,29,35,38^, wherefore their functionality remains to be conclusively established.

Cellular systems, in particular oocytes, are commonly used to study water transport through aquaporins^52^. While this allows water permeability to be determined without needing to isolate and purify the proteins, endogenous factors and integral membrane proteins may influence the results^53^. For this reason, studies in proteoliposomes are superior when it comes to determining and comparing the precise water transport through specific AQPs. In this work, we recombinantly expressed T125M, T126M and A147T and assessed their water permeability in proteoliposomes using stop-flow spectroscopy. Our studies show that all three mutants are able to form functional water channels with a water permeability that is 83.5%, 95.3% and 67.9% of wild-type AQP2 respectively (Fig. 4). For T125M and T126M, this is significantly higher than what was observed in oocytes, where the relative *Pf*-values were determined to be 25% (T125M) and 20% (T126M). In contrast, A147T had a lower *Pf* in proteoliposomes compared to oocytes where full water permeability was detected^29^. These differences are likely due to methodological differences and/or the presence of endogenous factors in the oocyte system.

Given that all three mutants were functional water channels, it is not surprising that CD-spectroscopy demonstrated that they are able to adopt the native AQP2 fold (Fig. 3a). Moreover, the crystal structures of T125M and T126M show that both mutants are structurally highly similar to wild-type AQP2 (Fig. 5). The AQP water pore runs through the middle of each monomer (Fig. 2) and as long as the overall fold of the protein allows the pore to form and the mutations does not cause any pore restriction or alter its chemical properties, the protein can be expected to be water permeable. This raises the question of why, if functional, are these mutants prevented from reaching the plasma membrane by the ER quality control system. In the case of A147T, this could be a result of low inherent protein stability, as shown by both CD spectroscopy and nanoDSF (Fig. 3). A reduced apparent stability for A147T has been demonstrated previously in cellular studies, where lower levels of A147T compared to wild-type AQP2 could be detected, suggesting an acceleration in degradation rate^25,29,30,50^. We show, for the first time, that the A147T mutation confers a significant reduction in stability of the protein itself. Given its location in the transmembrane region, such an effect on the inherent protein stability is not surprising. Moreover, it is interesting to note that this mutation lies in immediate proximity to a Cd^2+^-binding site found in the AQP2 crystal structure, which is presumed to represent a Ca^2+^-binding site of unknown function in vivo (Fig. 2e)^32^. It may be that the Ca^2+^-ion plays a stabilising role that is disturbed in the A147T-mutant. The low stability of A147T is also a probable reason for why this protein was difficult to reconstitute in proteoliposomes; we could only detect protein in one out of three reconstitution experiments. This is likely due to destabilization of the tetramer, since size-exclusion chromatography of the purified protein prior to the reconstitution experiment revealed that, in contrast to wild-type AQP2 and the other two mutants, A147T exists largely in a monomeric state (Supplementary Fig. S3). Nevertheless, the ability of A147T to form crystals in the same conditions and with similar morphology as wild-type AQP2 indicate that a small fraction of the protein are able to obtain the native tetrameric fold that is able to reconstitute into proteoliposomes. Since only tetrameric AQP2 can be expected to reach the plasma membrane, this could explain the observation that A147T displayed similar water permeability as wild-type AQP2 in oocytes^29^.

In contrast to A147T, a reduction protein stability can not explain the ER-retention of T125M and T126M. For both mutants, the stability is similar to wild-type AQP2, although nanoDSF suggests a slightly reduced stability for T125M. However, since the melting temperature determined by CD spectroscopy showed the opposite effect, T_m_ for T125M was higher compared to wild-type AQP2, this seems to be method-dependent. Moreover, the crystal structures of T125M and T126M reveal that the mutations do not confer any significant structural perturbations, not even around the mutation sites (Fig. 5). Thus, in order to understand the physiological consequences of these seemingly mild point-mutations, one may instead consider the glycosylation pathway which AQP2 undergoes on its route to the plasma membrane. Both mutations lie in the immediate proximity of an N-glycosylation site at N123, with T125 being part of the N-glycosylation recognition site (N − x − S/T, where x can be any residue except proline). Glycosylation at this site has been shown to not be a prerequisite for protein folding and tetramerization within the ER in MDCK-cells. However, while the non-glycosylated N123Q mutant was allowed to leave the ER, it was retained in the Golgi, suggesting that glycosylation plays an important role in post-ER sorting^27^. When expressed in oocytes, T125M was shown not to be glycosylated and was absent from the plasma membrane. This was proposed to be due to ER retention based on its intracellular localization^28^. However, the conclusion of whether a mutant is retained in the ER is typically drawn based on the appearance of a 32 kDa band in immunoblots, in addition to the non-glycosylated 29 kDa protein, representing the high-mannose glycosylated form of AQP2^27–31,34,38,50,51,54^. For T125M, this method is not applicable due to the lack of glycosylation. It seems plausible that, similar to N123Q, also this mutant is kept in the Golgi, after which it may be degraded via ESCRT-dependent targeting to lysosomes or the more recently identified Endosome and Golgi-associated degradation (EGAD) pathway^55^.

In contrast to T125M, T126M can be glycosylated and is therefore able to enter the glycosylation-dependent quality control pathway (Fig. 6). As described above, misfolded domains close to glycosylation sites can trigger reglucosylation by UGT, trapping the protein in the UGT-CNX/CRT cycle. In vitro studies have shown that UGT prefers substrates with hydrophobic residues down-stream to the N-glycan site (position 0)^56^. Specifically, a hydrophobic residue in position 3, as in the T126M mutant, correlated with increased glucosylation of glycopeptides. Hence, it seems plausible that T126M would be a better substrate for UGT than wild-type AQP2, despite being able to adopt the overall native fold. Moreover, it should be noted that UGT also monitors the quaternary structures of glycoproteins and has been proposed to recognize exposed hydrophobic residues in monomers that have failed to assemble into oligomers^57^. Since the tetramers of both A147T and T126M were less stable than wild-type AQP2 (Fig. S3), this may play a role in the ER-retention of both these mutants.

In conclusion, our work gives unique molecular insights into three AQP2-mutants that cause recessive NDI due to being retarded in the ER. The demonstration that the mutants are functional and able to retain the correct global as well as local fold illustrates that the degree of misfolding that is recognized by the ER/Golgi quality control systems can be very minor and that there are multiple factors that decide the fate of proteins with non-native sequences. The work presented here significantly increases our knowledge of the molecular mechanisms behind recessive NDI and suggests that it may be possible to design pharmaceutical therapies which aim to rescue functional mutants by promoting their targeting to the apical membrane.

## Methods

### Cloning

pPICZB encoding hAQP2 with an N-terminal 8 × histidine tag (His-tag) followed by a tobacco etch virus protease (TEV-protease) tag and a C-terminal truncation at P242^32^, were subjected to single-point mutagenesis using the QuickChange mutagenesis kit (Agilent Technologies) in order to introduce the T125M, T126M, and A147T mutations. The resulting plasmids were transformed into the *Pichia pastoris* X33-strain (Invitrogen) using electroporation. The transformed cells were plated on YPD with high zeocin concentration (2000 μg/ml) and well-growing colonies were cultured in small scale and screened for expression by analysis on Western blot utilizing the His-tag of the constructs for detection with anti-His antibodies (Takara Bio). The transformants with highest expression levels were used for larger scale expression and subsequent protein purification.

### Expression

Transformed *P. pastoris* containing the desired construct was streaked on yeast extract-peptone-dextrose-agar (YPD-agar) and grown at 30 °C for 3 days. A swab of cells was used to inoculate 100 ml of YPD-media and grown at 30 °C overnight. Once the culture reached an on OD600 of 25, it was used to inoculate a 3L fermenter (Belach Bioteknik), filled with 1.5 l basal salt media (BSM) The cells were first allowed to grow by feeding them glycerol containing 1.5% Pichia Trace Minerals (PTM) for 24 h, followed by induction of the AOX1 promoter by switching to methanol feeding (also containing 1.5% PTM) for 36 to 48 h. The cells were then harvested and spun down at 6,000 rpm, and stored at − 20 °C.

### Purification

The frozen cells were thawed in breaking buffer (50 mM KPi pH 7.5, 5% glycerol, 2 mM ethylenediaminetetraacetic acid (EDTA) at room temperature and broken using 0.5 mm glass beads in a BeadBeater (Biospec). 50–100 g of cells were resuspended in 300 ml breaking buffer (50 mM KP_i_ pH 7.5, 5% glycerol, 2 mM EDTA, 1 mM PMSF) were put through 12 × 30 s cycles of bead beating, with 30 s between each cycle. The broken cells were spun down at 10,000 g for 1 h at 9,500 rpm. The supernatant, containing the membranes, were then centrifuged at 100,000 g for 1 h at 45,000 rpm. The pelleted membranes were homogenized (Potter–Elvehjem, Wheaton USA) in urea buffer (50 mM Tris-HCl pH 9.5, 4 M urea, 2 mM EDTA), and centrifuged for 2 h at 45,000 rpm. The second pellet was homogenized in membrane buffer (50 mM Tris-HCl pH 8, 20 mM NaCl, 10% glycerol) with 1 mM phenylmethane sulfonyl fluoride (PMSF) and 2 mM EDTA, and spun down for 1 h 15 min at 45,000 rpm. The final pellet was weighed and resuspended in < 2 ml/g membrane buffer and flash frozen in liquid nitrogen and stored at − 80 °C.

8 g of thawed membranes were diluted with membrane buffer to 25 ml after which 25 ml solubilization buffer (20 mM Tris-HCl ph8, 300 mM NaCl, 4%OGNG, 1 mM PMSF) was added drop by drop, while stirring at 4 °C. The membranes were solubilized at 4 °C for 1 h, after which 10 mM imidazole was added and the sample was centrifuged for 30 min at 50,000 rpm. The supernatant was loaded onto an IMAC column (HisTrap HP 5 ml, GE Life Sciences), equilibrated with Buffer A (20 mM Tris–HCl pH 8, 300 mM NaCl, 0.2% OGNG) + 10 mM imidazole, using a Superloop (Cytiva) and a flow rate of 1 ml/min at 4 °C. Stepwise elution was performed by combining buffer A with buffer B (20 mM Tris-HCl, 300 mM NaCl, 300 mM imidazole, 0.2% OGNG) at different ratios. Specifically, after washing the column with 2 column volumes (CV) with 10 mM imidazole, followed by 5 CV with 75 mM imidazole, the target protein was eluted with 300 mM imidazole. Fractions were collected throughout the purification and analyzed through SDS-PAGE and Western Blot. The fractions containing the desired protein were combined, concentrated to 2.5 ml (molecular weight cut-off at 30 kDa, Vivaspin), and passed through a Sephadex G-25 PD-10 desalting column (GE Life Sciences), and eluted with 3.5 ml buffer A.

The His-tag was removed by adding His-tagged Tobacco Etch Virus (TEV)-protease^58^ in TEV cleavage buffer (0.5 mM tris(2-carboxyethyl) phosphine (TCEP), 20 mM Tris-HCl pH 9.5) at a 2:1 ratio to the amount of protein in the sample. The reaction mixture was left to incubate 4 °C overnight on a table rotating around the horizontal axis. The following day, the cleaved fragment and the TEV-protease were removed by a second IMAC. Imidazole was added to the reaction to a final concentration of 20 mM, after which it was filtered through a 0.45 µm sterile filter. The sample was loaded onto a HisTrap HP column (equilibrated with buffer A + 20 mM imidazole), and the cleaved protein was isolated by collecting the flowthrough. A final wash was run with 300 mM imidazole in order to elute TEV-protease and cleaved fragments from the column.

The flowthrough fraction containing the desired protein were concentrated to < 500 µl and passed through a spin filter. The sample was then injected on a SEC column (Superdex 200 Increase 10/300 GL, GE Life Sciences), equilibrated with buffer A, at 4 °C and the peak fractions were collected and analysed on SDS-PAGE. The desired fractions were concentrated to 10 mg/ml, after which glycerol was added to the sample to a final concentration of 5%. The sample was flash frozen, and stored at − 80 °C.

### Circular dichroism spectroscopy

Samples were diluted to 0.2 mg/ml with a buffer containing 10 mM KP_i_ pH 7.5, 100 mM NaCl, 0.2% OGNG and 5% glycerol and added to a cuvette with a pathlength of 1 cm. CD spectra between 250 and 200 nm were obtained in a Jasco J-720 spectrometer (Jasco, Easton, Maryland, USA) at 20–95 °C with a scanning speed of 50 nm/min and a data integration time (DIT) of 4 s. Each scan was recorded in triplicates. The temperature was controlled using a built-in Peltier-controller. The temperature was raised 1 °C per minute, until the temperature was 5 °C above the previous reading, with a 10 s delay upon reaching each new temperature level.

The spectra were extracted and processed using Pandas (version 1.4.2), Numpy (version 1.25.5), Matplotlib (version 3.5.1), and Scipy (version 1.7.3) packages of Python (3.9.12). The ellipticity was converted to mean residual ellipticity (MRE) using Eq. 1:

1 $$MRE=\frac{\theta *MRW}{10*P*C}$$

where MRW is the mean residual weight (molecular weight of the protein divided by the number of residues), T is the temperature, θ_conv_ is the ellipticity conversion factor (3298), *P* is the pathlength of the cuvette (0.1 cm), and C is the concentration (0.2 mg/ml).

Curves were fitted using Eq. 2,

2 $$\theta =\frac{\left(T{\alpha }_{n}+{\beta }_{n}\right)+\left(T{\alpha }_{d}+{\beta }_{d}\right){e}^{\frac{\Delta G}{RT}}}{1+{e}^{\frac{\Delta G}{RT}}}$$

where ${\alpha }_{n}$ and ${\alpha }_{d}$ are the native and denatured states at 0 K respectively, ${\beta }_{n}$ and ${\beta }_{d}$ are the slopes with respect to temperature of the native and denatured states respectively, *R* is the ideal gas constant, and $\Delta G$ is the free energy of unfolding. Given these conditions, we may calculate the melting temperature (${T}_{m}$) using the Gibbs–Helmholtz equation, assuming that the heat capacity of the protein, $\Delta {C}_{p}=0$;

3 $$\Delta G=\Delta H\frac{1-T}{{T}_{m}}$$

where $\Delta H$ is the enthalpy, $T$ is the measured temperature, and ${T}_{m}$ is the melting temperature.

### Nano differential scanning fluorometry

Measurements were performed in triplicates on a Prometheus NT.48 (NanoTemper), at 0.2 mg/ml, between 20 and 95 °C. Data was exported using PR.ThermControl v2.0.4 (NanoTemper), and fitted using Eq. 2 as described above. T_onset_ was calculated using MoltenProt at the eSPC online data analysis platform ^37,59^.

### Stopped-flow spectroscopy

*E. coli* polar lipids from Avanti were dehydrated using a weak nitrogen stream (4 h). Subsequently, the lipids were resuspended in 20 mM Tris-HCl pH 8, 200 mM NaCl and 10 mM 5(6)-carboxyfluorescein. The lipids were rehydrated to a final concentration of f 20 mg/ml. The lipid suspension was sonicated in a sonication bath for 3 × 15 min, with 5 min breaks between cycles, after which the lipids were frozen in liquid nitrogen and thawed three times. Once thawed three times, the lipids were passed through first a 200 nm polycarbonate filter 11 times, then a 100 nm polycarbonate filter 11 times, using an extruder (Mini-Extruder, Avanti). The lipids were diluted to 4 mg/ml with reconstitution buffer (20 mM Tris–HCl pH 8, 200 mM NaCl) supplemented with 25% glycerol and 0.2% OGNG. Additionally, Triton X-100 was added to the sample to a final concentration of 0.175%. AQP2 was added to the lipid suspension using a lipid-to-protein-ratio (LPR) of 100. Each sample was dialyzed overnight at 4 °C in reconstitution buffer. The samples were centrifuged at 57,000 × g for 1.5 h, and the resulting pellets were resuspended in reconstitution buffer.

The shrinkage assay was performed on an SX-20 Stopped-Flow Spectrometer system (Applied Photophysics), where the liposomes were rapidly mixed with reaction buffer with 570 mOsm sucrose. Data were collected at 495 nm at a 90° angle for 10 s. All data were collected at 20 °C. Empty liposomes were used as a negative control.

Data for each sample was collected 10 times, after which the data was normalized and averaged. The average was then fitted using Eq. 4,

4 $$y={y}_{0}+{A}_{1}{e}^{-{k}_{1}\left(x-{x}_{0}\right)}+{A}_{2}{e}^{-{k}_{2}\left(x-{x}_{0}\right)}$$

where *A* is the amplitude of the scattering, while *k* is the swelling rate of the liposomes once exposed to the osmotic pressure. *A*_*1*_ and *k*_*1*_ are assumed to be representative of the passive water transportation conducted via AQP2, whereas *A*_*2*_ and *k*_*2*_ are assumed to be representative of the passive diffusion across the lipid membrane. The data processing and subsequent curve fitting were performed using using Pandas (version 1.4.2), Numpy (version 1.25.5), Matplotlib (version 3.5.1), and Scipy (version 1.7.3) packages of Python (3.9.12).

Once fitted, k_*1*_ was used to calculate the permeability (*Pf*) of the samples, using Eq. 5,

5 $$Pf=\frac{{k}_{1}}{\frac{{S}_{0}}{{V}_{0}}{V}_{w}{C}_{out}}$$

where *S*_*0*_ is the initial surface area of the liposomes, *V*_*0*_ is the initial surface volume, *V*_*w*_ is the partial molar volume of water (18 cm^3^/mol), and *C*_*out*_ is the external osmolality (285 mOsm). With the exception of A147T which could only be successfully reconstituted in one preparation, *Pf* was calculated from the average of 10 measurements for three individual proteoliposome preparations.

In order to adjust for differences in reconstitution levels, the amount of protein in the proteoliposomes was analysed by Western blot using antibodies directed against AQP2 (SantaCruz, AQP2-H40, sc-28629). In each lane, 5 μl of the liposome preparation was added. The intensity of the bands were quantified using the online software ImageJ^60^, resulting in a normalization factor that was used to calculate the final *Pf*.

### Analysis of size-exclusion chromatograms

Each chromatogram was normalized by setting the baseline as 0 and the maximum peak height as 1. The integral of each peak was percentually quantified in relation to the sum of the integrals of the total population. Based on this, the amount (in %) of individual oligomeric states in the sample was estimated.

### Crystallization and structural determination

Crystallization trials were set up using the hanging drop method. 0.5–1 μl of protein solution (10 mg/ml) was mixed with an equal amount of reservoir solution (20–30% PEG400, Tris-HCl pH 8.5, 0.1 M MgCl_2_, 0.1 M NaCl) to which 0.1 M CdCl_2_ had been added in a 1:4 ratio, and left to equilibrate at room temperature or 4 °C. Rod-like crystals grew to full size within a few days and were mounted in cryo loops (Molecular Dimensions) and flash frozen in liquid nitrogen. Crystals grown at < 24% PEG400 were cryo-protected by soaking in reservoir solution + 30% PEG400, otherwise no cryo-protection was used. Crystals were screened for diffraction quality at Deutches Elektronen-Synchrotron (DESY) PETRA-III (beamline P13), European Synchrotron Radiation Facility (ESRF) beamline ID29, and MAX IV (BioMAX). T125M grown in 20% PEG_400_ at 6 °C, T126M grown in 24% PEG_400_ in room temperature, and A147T grown in 22% PEG_400_ at 6 °C produced the best diffraction. Final data sets for T125M and T126M were collected at the MAX IV BioMAX beamline^61^ and PETRA-III, beamline P13^62^, respectively.

The T126M data was processed using MOSFLM^63^ and scaled using AIMLESS of the CCP4 suite^64^. For T125M, two data sets from two different crystals were processed in XDS and merged and scaled using XSCALE^65^. The final resolution for the two data sets were 3.15 Å and 3.9 Å for T126M and T125M respectively. Molecular replacement was performed in PHASER^66^ using the previously known structure of AQP2 (PDB code 4NEF) as a model^32^. Refinement was done in Phenix^67^.

### Statistical evaluation

T_m_, T_onset_ and *Pf* -values are presented as means ± standard deviation. The statistical significance of the difference was estimated using a Z-test for population means. Differences were considered significant if the *p*-value was less than 0.05

$$Z=\frac{\left(\overline{{X }_{1}}-\overline{{X }_{2}}\right)}{\sqrt{{\sigma }_{1}^{2}+{\sigma }_{2}^{2}}}$$

### Supplementary Information

Supplementary Information.

## Data Availability

Structure coordinates and structure factors have been deposited in the Protein Data Bank (PDB). All other data can be made available from the corresponding author upon reasonable request.
