# Supplementary material for: Structural and functional analysis of aquaporin-2 mutants involved in nephrogenic diabetes insipidus
Source: Sci Rep. 2023 Sep 6;13:14674. doi: 10.1038/s41598-023-41616-1 (PMC10482962; doi:10.1038/s41598-023-41616-1)
Supplement: Supplementary file 1 — Supplementary Information. [file 41598_2023_41616_MOESM1_ESM.pdf]

Structural and functional analysis of aquaporin-2 mutant involved in nephrogenic diabetes insipidus

Carl Johan Hagströmer[1], Jonas Hyld Steffen[2] Stefan Kreida[1], Tamim Al-Jubair[1], Anna Frick[3], Pontus Gourdon[2,4] Susanna Törnroth-Horsefield[1*]

Table S1: T_m and T_{onset} for the individual constructs, measured through circular dichroism (CD) and nano differential scanning fluorometry (nDSF). Values are given as means ± standard deviation. Statistical significance between wild-type AQP2 and the mutants is indicated by the p-value.

	**CD**		**nDSF**			
	T_m (°C)	p-value	T_m (°C)	p-value	T_{onset} (°C)	p-value
Wild-type	71.03 ± 0.16		70.39 ± 0.27		61.60 ± 0.41	
T125M	73.45 ± 0.65	0.00015	65.27 ± 0.21	<0.00001	54.70 ± 0.21	<0.00001
T126M	72.14 ± 0.25	0.00092	67.87 ± 0.39	<0.00001	59.56 ± 0.35	0.000077
A147T	61.71 ± 0.96	<0.00001	59.71 ± 0.34	<0.00001	51.22 ± 0.64	<0.00001

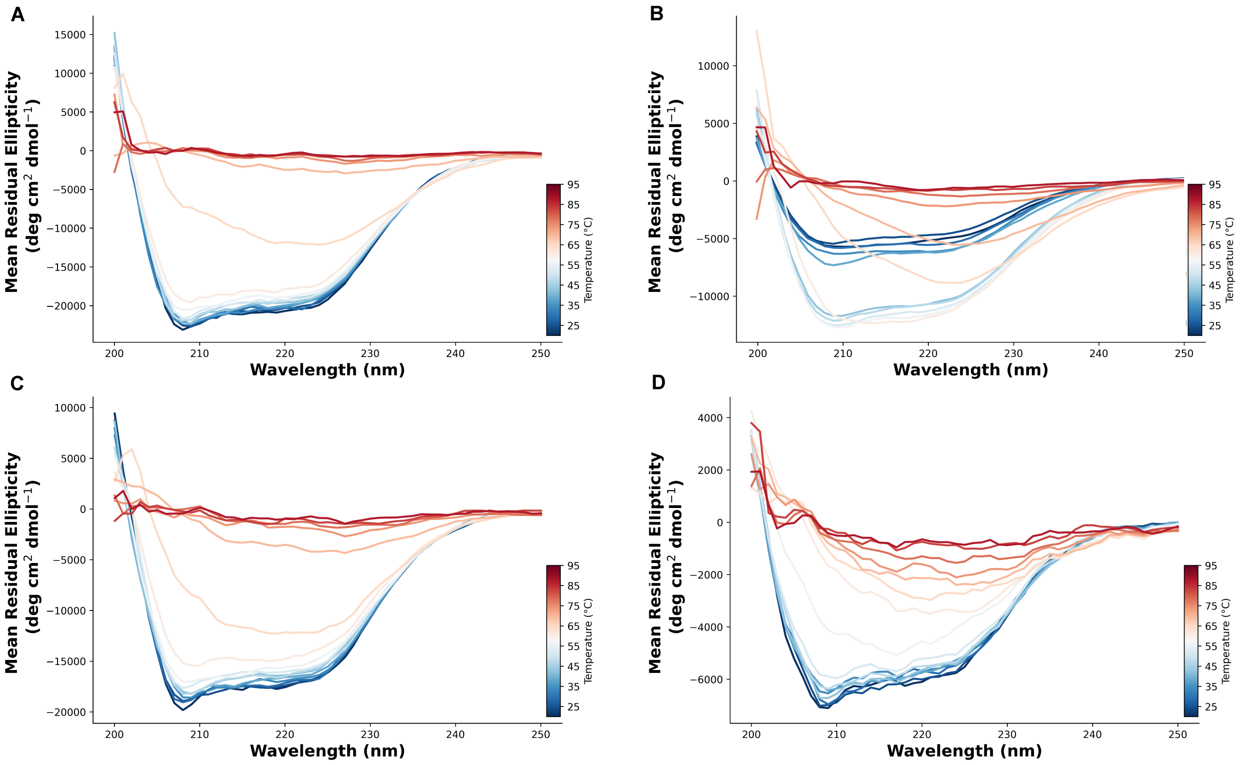

Figure S1. Circular dichroism data measured from 200nm to 250nm, at 20 to 95°C using 5°C intervals, of wt AQP2 (A), T125M (B), T126M (C), and A147T (D).

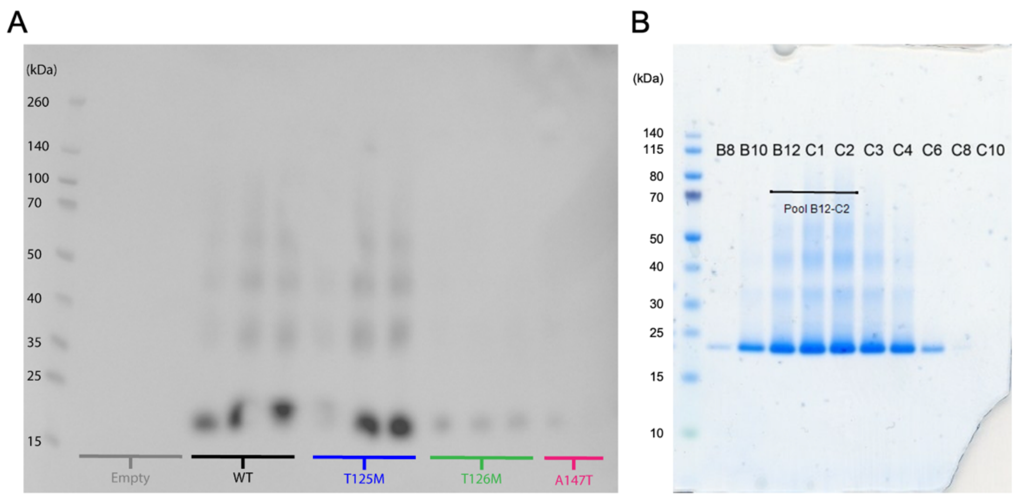

Figure S2. A. Western blot of proteoliposomes using an antibody directed against AQP2 (AQP2-H40, Santa Cruz Biotechnologies) for detection. Each lane was loaded with 5 µl of the liposome preparation. B. Typical SDS-PAGE gel for purified AQP2 showing the characteristic band pattern that is also observed on the Western blot. Fractions from size-exlusion chromatography are indicated with B8-C10.

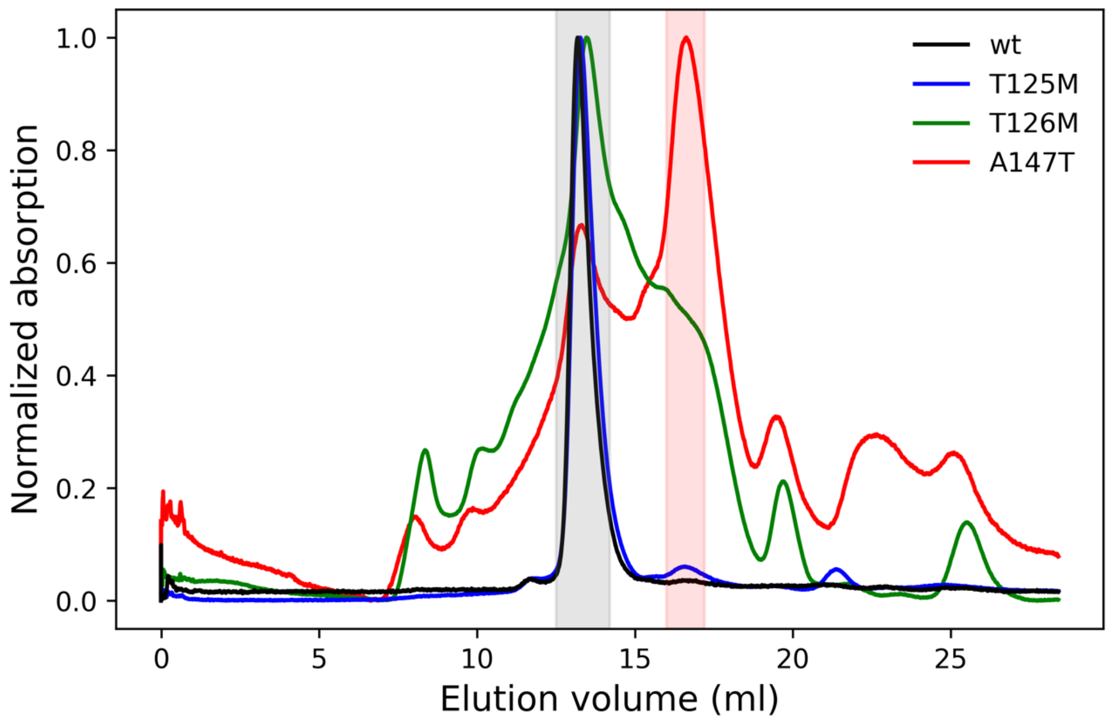

Figure S3. Size exclusion chromatography profiles of wt AQP2 (black), T125M (blue), T126M (green), and A147T (red). The grey and red columns highlight fractions corresponding to the tetrameric and monomeric states of the protein. The y-axis corresponds to normalized absorption at 280 nm.

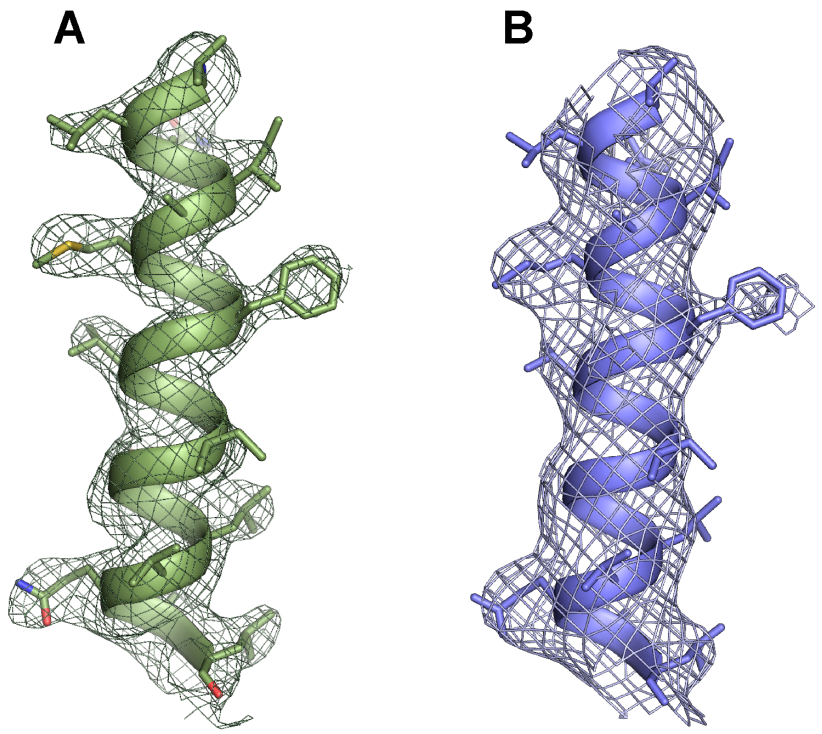

Figure S4. Typical 2Fo-Fc electron density for T126M (A, green) and T125M (B, blue), contoured at 2σ.
